# Comparison of BIOBALL and EZ-Accu Shot for accurate quantification as certified quality control reference material

**DOI:** 10.1128/jcm.00520-25

**Published:** 2025-07-01

**Authors:** Dustin A. Baird, James E. T. Gebo, Anna F. Lau

**Affiliations:** 1 Sterility Testing Service, Department of Laboratory Medicine, Clinical Center, National Institutes of Health 24481, Bethesda, Maryland, USA; Endeavor Health, Evanston, Illinois, USA

**Keywords:** quality, certified, quantitation, GMP, COA, reproducibility, accuracy

## LETTER

Product sterility testing and other Good Manufacturing Practices (GMP) testing services, such as environmental monitoring, aseptic processing simulations, and glove fingertip testing, continue to be pushed onto clinical microbiology laboratories that support hospital pharmacies and cell therapy manufacturing facilities. Differences between clinical and GMP microbiology laboratories are discussed elsewhere (1, 2). Here, we focus on quality control (QC) testing of culture media where clinical and GMP requirements differ substantially (Table 1). This study is relevant for any clinical laboratory that supplies or uses culture media for GMP testing where regulatory agencies expect the use of quantified and specified QC organisms for media qualification studies prior to media release (Table 1).

**TABLE 1:** Differences in the practice of media QC for clinical and cGMP applications

	Media QC for clinical practice	Media QC for GMP tests	
Regulatory authority	Centers for Medicare and Medicaid Services under the Clinical Laboratory Improvement Amendments Act.	United States Food and Drug Administration under the Food, Drug, and Cosmetic Act.	
Regulatory requirement	CAP checklist MIC.11035, MIC.11038, MIC.11045, and MIC.11055. Commercially prepared media are regulated under 21 CFR 820.	USP chapters <61>, <62>, <71>, and <1117> (3–6). 21 CFR 211.160, 211.167, and 211.194 for drugs. 21 CFR 610.12 for biologics. FDA Guidance for Industry (7). Commercially prepared media are regulated under 21 CFR 211.	
Exemptions	May be exempt via an Individualized Quality Control Plan.	No exemptions.	
Growth promotion			
Number of organisms to be tested	As instructed in CLSI M22 (8).	As instructed in USP <61>, <62>, and <71> (3–5).	
Method	Prepare a 0.5 McFarland suspension, dilute 1:100 (for non-selective media) or 1:10 (for selective media). Culture 10 µL equivalent to 10^3^–10^4^ CFU/plate for non-selective media and 10^4^–10^5^ for selective media. Incubation conditions as defined in CLSI M22 (8).	Inoculate <100 CFU, in duplicate, onto the respective media. Include inoculation of previously qualified media, in duplicate, for QC comparison. Incubation conditions as defined in USP <61>, <62>, and <71> (3–5).	
Acceptability criteria	Qualitative results. Confirmation of growth with colonies of sufficient size and characteristic morphology.	Quantitative results. 50%–200% recovery (average test plate CFU [new lot] divided by average colony count plate CFU [existing lot], multiplied by 100) (9).	
Sterility check			
Sample size	Optional for commercially prepared media. No sample size defined. Clinical labs have been known to pull a single plate only for sterility checks.	Optional for commercially prepared media, but considered best practice for verification of the COA. ASQ/ANSI Z1.4-2003 (R2018) is recognized by the FDA and can be used to determine appropriate statistical volume based on batch size (10).	
		**Batch size in units**	**Sample size in units**
		1–25	3
		26–90	5
		91–150	8
		151–280	13
		281–500	20
		501–1,200	32
		1,201–3,200	50
Incubation conditions	35°C for ≤72 hours.	Temperature as defined in USP <61>, <62>, and <71> for ≥5 days for solid media and ≥14 days for liquid media.	
Acceptability criteria	No bacterial or fungal growth.	Terminally sterilized media: no growth. Non-terminally sterilized media: no industry standard. ≤2 plates have growth, and ≤3 CFU across both plates is used in our laboratory for best practice based on industry experience.	

After a recent trend of low CFU (≤10 CFU) recoveries and failed growth promotion tests using Microbiologics EZ-Accu Shot, we undertook a study to assess the reproducibility and accuracy of quantified organisms compared with the published certificate of analysis (COA) for the EZ-Accu Shot and the bioMérieux BIOBALL, both marketed as certified reference material for use in the GMP industry. Organisms were rehydrated following the manufacturer’s instructions, cultured onto various media, and colony counts were obtained within the specified incubation period (Table 2). A total of 2,584 EZ-Accu Shot tests (covering 204 product lots) were conducted, but only 1,764 tests were analyzed (from 153 lots) because 820 tests (32%) had statistical errors reported in the COA (affecting 51 lots). These errors, confirmed by the vendor in personal communications, included incompatible statistical calculations that mixed data from entire vial volume (1.1 mL) measurements with aliquot volume data (100 µL), which affected product lots released between 2016 and 2018. A total of 230 BIOBALL tests across 26 lots were analyzed.

**TABLE 2:** Summary of results comparing quantification results of EZ-Accu Shot and BIOBALL against the manufacturer’s COA

Category	Organisms	EZ-Accu Shot	BIOBALL
Organism concentration claims in the manufacturer’s COA		10–100 CFU per inoculum	50 CFU per inoculum
No. of lots evaluated	*Bacillus spizizenii^a^*	24 (was 33 but 9 removed from study^d^)	3
	*Staphylococcus aureus^a^*	28 (was 35 but 7 removed from study^d^)	5
	*Pseudomonas paraeruginosa^a^*	26 (was 34 but 8 removed from study^d^)	4
	*Clostridium sporogenes^a^*	15 (was 21 but 6 removed from study^d^)	5
	*Candida albicans^b^*	32 (was 42 but 10 removed from study^d^)	5
	*Aspergillus brasiliensis^b^*	28 (was 39 but 11 removed from study^d^)	4

		As published in COA	Adjusted to correct calculation errors^c^	As published in COA
Percentage of tests within 95% of the published confidence interval	All organisms	7%	49%	82%
	*Bacillus spizizenii*	5%	58%	100%
	*Staphylococcus aureus*	13%	72%	100%
	*Pseudomonas paraeruginosa*	3%	26%	96%
	*Clostridium sporogenes*	0%	25%	94%
	*Candida albicans*	8%	48%	97%
	*Aspergillus brasiliensis*	8%	45%	33%
Range of standard deviation (SD) across product lots compared with the manufacturer’s published mean in the COA	*Bacillus spizizenii*	−6 to +7 SD → from 24 lots, 265 tests		−2 to +2 SD → from 3 lots, 20 tests
	*Staphylococcus aureus*	−6 to +6 SD → from 28 lots, 304 tests		−2 to +2 SD → from 5 lots, 34 tests
	*Pseudomonas paraeruginosa*	−2 to +9 SD → from 26 lots, 276 tests		−3 to +2 SD → from 4 lots, 28 tests
	*Clostridium sporogenes*	−3 to +10 SD → from 15 lots, 40 tests		−3 to +1 SD → from 5 lots, 32 tests
	*Candida albicans*	−6 to +8 SD → from 32 lots, 448 tests		−3 to +3 SD → from 5 lots, 62 tests
	*Aspergillus brasiliensis*	−5 to +8 SD → from 28 lots, 431 tests		−5 to −1 SD → from 4 lots, 54 tests

^a^ Bacteria inoculated onto tryptic soy agar (TSA), TSA with lecithin and Tween 80 (TSALT), and TSA with 5% Sheep Blood (TSASB) incubated at 30°C–35°C for 24–72 hours as per USP <61>, <62>, and <71>.

^b^ Fungi inoculated onto TSA, TSALT, TSASB, Sabouraud dextrose agar (SDA), and SDA with lecithin and Tween 80 (SDALT) incubated at 20°C–25°C for 48–120 hours as per USP <61>, <62>, and <71>.

^c^ Recalculation of the 95% CI was performed independently using the published mean ± 1.96 SD data in the Microbiologics COA. The manufacturer confirmed that there was a calculation error in their software that affected all COAs released for multiple organisms and multiple lots over a long time period.

^d^ Tests from a total of 51 COAs were excluded due to incompatible statistical calculations that mixed data from entire vial volume with aliquot volume.

As highlighted in Table 2, the accuracy and reproducibility of organism quantification compared with the manufacturer’s COA were significantly better for the BIOBALL compared with the EZ-Accu Shot across all six organisms and numerous product lots (26 lots for BIOBALL and 153 lots for EZ-Accu Shot). Variations in test conditions (media, incubation conditions, and product expiration date) did not impact results (data not shown). Importantly, an independent review of EZ-Accu Shot COAs showed that all COAs issued by Microbiologics had 95% confidence interval ranges that were too narrow, possibly explaining why only 7% of tests (range 0%–13%) fell within the manufacturer’s published COA (Table 2). The manufacturer confirmed that these errors were a result of incorrect formulas used in their software (personal communications). Nevertheless, recalculation of the 95% confidence interval using the published mean ± 1.96 standard deviation showed that only 48% of tests (range 25%–72%) fell within the 95% confidence interval. This, together with the (i) calculation errors (51/204 COAs) identified earlier in the 32% (*n* = 820) tests that were excluded from the data set, and (ii) the wide standard deviation (−6 to +10) observed over 1,764 tests (Table 2), suggests quality concerns for the EZ-Accu Shot. In contrast, data from 230 BIOBALL tests showed better reproducibility and tighter alignment with the manufacturer’s COA, reflecting a higher level of quality standards through the manufacturing process and COA generation (Table 2).

Commercially prepared media intended for use in the GMP setting are regulated under 21 CFR Part 211. Reference material used to qualify media should consistently meet high-quality standards for reproducibility and align with statistical data certified in the COA. Furthermore, formula calculations used in software for COA generation should be accurate. Laboratories involved in providing media for GMP testing are reminded that sole reliance on a manufacturer’s COA is not best practice, despite manufacturer claims of “certification” and “quality.” Instead, materials (even those with COA) should be independently verified by the laboratory, results should be tracked and trended, and negative trends should be investigated and reported immediately.
